# Digital health literacy among older adults in China: a cross-sectional study on prevalence and influencing factors

**DOI:** 10.3389/fpubh.2025.1661177

**Published:** 2025-09-23

**Authors:** Xingxia Zhang, Yongqing Yuan, Jie Jiang

**Affiliations:** West China Hospital, Sichuan University, Chengdu, China

**Keywords:** digital health literacy, e-health literacy, digital divide, older adults, internet, China

## Abstract

**Background:**

The rapid integration of digital technologies into healthcare has emphasised the importance of digital health literacy (DHL) in enhancing health outcomes. Despite the increasing adoption of the internet among older adults in China, disparities in eHealth literacy persist, necessitating urgent investigation.

**Objective:**

This study aimed to investigate the status and predictors of DHL among older adults in Sichuan Province, China.

**Methods:**

A cross-sectional survey was conducted from October to December 2024 using a multistage stratified sampling method. Data were collected using the Chinese version of the eHEALS questionnaire. Univariate and multivariable binary logistic regression analyses were performed to identify predictors of DHL, adjusting for sociodemographic, health-related, and internet use variables.

**Results:**

A total of 1,202 valid responses from adults aged 60 years and over were analysed. Only 30.45% (*n* = 366) of participants met the eHEALS threshold (mean total score: 22.30 ± 10.62). Sociodemographic factors (residence and gender), internet behaviours, and training experience were all significantly associated with DHL among older adults.

**Conclusion:**

This study reveals suboptimal digital health literacy among older adults in China, driven by rural–urban disparities, gender gaps and limited digital engagement. In future, interventions tailored to specific needs, such as community-based training, telemedicine promotion and family-supported digital education, will be critical in bridging this gap.

## 1 Introduction

Digital technology has become deeply integrated into the medical and health industry. Technological advances and industrial transformation have driven the transition of traditional healthcare service models to a new phase of digital health (1). Digital health literacy (DHL) is a new interdisciplinary research field that has garnered widespread academic attention (2). DHL (eHealth literacy) refers to the ability of individuals to search, filter, understand, evaluate and apply health information obtained through electronic platforms to solve health-related issues (3). Studies have proven that digital health literacy is positively correlated with health-related behaviours (4), self-health management (5), quality of life (6) and disease-related knowledge and engagement (7). It is an essential competency for promoting public health and health management (8). Digital divide persists especially among older individuals, despite the broad accessibility of mobile tools (9). It is vital that greater attention is paid to the DHL of older people, who are known as “digital refugees” (10) in the digital era.

The global population ageing issue is accelerating worldwide. Projections indicate an increase in the proportion of the global population aged 65 and above from 6.8% in 2000 to 14.3% by 2040, signifying the transition to a moderately ageing society (11). By the late 2070s, the United Nations projects that the population of individuals aged 65 and over will exceed that of those under the age of 18 (11). China is currently home to the world’s largest older population and is experiencing the fastest aging rate. Indeed, China is home to 1/5 of the world’s older people (12), with 80.0% of older adults self-reporting chronic health conditions (13). The enhancement of DHL among the older population is recognised as one of the most fundamental, cost-effective, and efficient measures to improve overall population health outcomes due to the advantages of reaching large numbers of people at relatively low cost (14). The internet can provide extensive, readily accessible, and cost-effective health-related information to users (15). This enables individuals to easily access online health information and empowers them to make informed decisions and take proactive control of their health management (16, 17). The 55th Statistical Report on Internet Development in China revealed that the number of internet users aged 60 years and older grew from 7.3 million in 2009 to 157.25 million in 2024 (18). This indicates that the internet is making further inroads into the middle-aged and older population, and that internet use is an important means of accessing health-related information (19). However, older people often fail to seek, understand or apply the online health information because of their limited digital knowledge, skills and support (20–22). Research has indicated that individuals with low health literacy are less likely to use the internet to obtain health or medical information (Odds Ratio [OR] = 0.60 [95% CI 0.47–0.77]), only 9.7% of older individuals with low health literacy use the internet to obtain health information, whereas 31.9% of those with adequate health literacy among older Americans do so (23).

A scoping review revealed that research on DHL in older adults is still in its infancy (20), especially in China, the current level of digital health literacy among older adults still to be determined. A few studies investigated the level of older adults in Beijing (24), Chongqing (25), Luoyang and Zhengzhou (26). As of current data, Sichuan Province has an older population aged 60 and above reaching 18.164 million, accounting for 21.7% of its total population (27). This places Sichuan as the third most aged region nationally, marking its entry into a deep aging society (27). However, no publication related to DHL of older adults in Sichuan has been searched. In addition, Subjective factors (such as attitudes toward Internet health information) and social environment factors (family and social support) have received insufficient attention, leading to research that lacks systematicity and comprehensiveness (25). Therefore, the study takes Pengzhou in Sichuan Province as an example to conduct a cross-sectional survey. The aims of this study are: (1) Use the eHealth Literacy Scale (eHEALS) to assess the status quo of DHL among the older adults; (2) Analyse the influencing factors of DHL of older adults by comprehensively considering the sociodemographic characteristics, attitude towards Internet health information, habits of Internet usage, and external support systems; (3) Provide a reference for the formulation of improvement strategies and the development of intervention studies in the future research.

## 2 Methods

### 2.1 Study design

#### 2.1.1 Inclusion and exclusion criteria

A cross-sectional survey was conducted in Pengzhou City, Sichuan Province. The absence of granular demographic data for the older population (aged ≥60 years) at the sub-district (town) level in Pengzhou rendered the construction of a precise sampling frame specifically targeting older adults unfeasible. Consequently, a city-wide sampling framework covering the general adult population was adopted.

The survey participants were comprised of permanent residents aged ≥18 years (defined as individuals residing in the city for ≥6 months, irrespective of their household registration status). Participants were required to possess: (1) Basic comprehension capacity sufficient to understand survey items; (2) Ability to complete questionnaires or provide verbal responses; (3) Willingness to provide informed consent. Participants were excluded if they met any of the following conditions: (1) Severe visual, auditory, or verbal communication impairments; (2) Significant cognitive dysfunction or physical comorbidities precluding survey participation; (3) Institutionalized populations; (4) Inability to complete the study for other investigator-determined reasons, such as illiterate or functionally impaired participants whole is unable to understand the means of the questionnaire.

In view of the study’s primary objectives, data analysis was restricted to participants aged ≥60 years (*n* = 1,202).

#### 2.1.2 Sampling methods

A stratified cluster sampling design was implemented. The target population was first stratified into mutually exclusive sub-districts/townships as sampling strata. Proportional allocation was applied to determine the sample size per stratum, where each sub-district/township was assigned a sample size proportional to its population share relative to the total population. Within each stratum, communities or administrative villages were randomly selected as primary sampling units using simple random sampling. A complete enumeration was then conducted within all sampled clusters, enrolling all eligible individuals residing in the selected communities/villages.

#### 2.1.3 Sample size calculation

The sample size was calculated according to the sample size calculation formula for the limited population of the cross-sectional survey:

$$n_{1}={\left(\frac{Z_{\alpha /2}}{\delta }\right)}^{2}*(1-P)*P$$

$$n_{2}=\frac{n_{1}*N}{n_{1}+N}$$

$Z_{\alpha /2}$ was 1.96 (*α* = 0.05, two-sided), *δ* = 2% (allowable absolute error), and the maximum *p* value was 0.5. Considering a rejection rate of 20%, the final sample size was 2,913. The details were presented in Supplementary materials. According to the ratio of the local older population, the proportion of older people in the sample should be no less than 25.28% to ensure an effective sample size.

### 2.2 Methods of survey

#### 2.2.1 Survey tools

The questionnaire was developed by the research group based on existing assessment tools and previous related researches. The questionnaire comprises two parts. The first part covers demographics of residents, including age, gender, location, marital status, ethnic group, education, occupation, family numbers, domicile, monthly household income per capita, residential status, marital status, self-rated health status, medical insurance, degree of health concerns and chronic diseases (25, 28, 29). It also covers internet usage habits, attitudes, and skills, which have been investigated in previous studies (25, 30, 31). Two performance-based items including “bookmark function” and “health website overview” were included to assess the Internet knowledge of individual according to the Digital Health Literacy Instrument (DHLI) (32). Social support (such as Children’s concern about their health, experience of DHL training) were included because the digital health divide is likely determined not only by DHL of individuals but also by various other interacting factors, such as the individual lifestyle factors, attitudes, social and community networks, and the cultural and environmental conditions (33–35). The second part is the Chinese version of the eHEALS, proposed by Norman and H. Skinner in 2006 (3), which was culturally adapted and validated for Mandarin-speaking populations by Guo et al. (36). It is an eight-item measure of eHealth literacy that was developed to evaluate consumers’ knowledge, comfort level, and perceived ability to find, evaluate, and apply electronic health information to health problems. It uses a Likert five-level scoring method, with scores ranging from one to five, and the total score ranges from eight to 40 points. The Chinese version scale has high reliability (Cronbach’s α = 0.913) which is the most widely used tool to measure digital health literacy among the older (37). Higher scores indicate greater e-health literacy. Scores ≥32 classified as “qualified”—maintaining the original threshold to facilitate international comparisons (3, 38). Through iterative focus group discussions within the research team, we refined and finalized the survey questionnaire. The complete questionnaire is available in the Supplementary materials.

#### 2.2.2 Data collection

Research group members and community health workers who had received standard training served as investigators, explaining the purpose and significance of the research to the participants. The Wenjuanxing platform was used to survey and collect data, which is the most popular digital survey platform in China (39). Questionnaires were considered valid only if all the included questions were answered according to our predefined validation criterion. This study was conducted from October to December 2024 using dual-mode data collection approach comprising both online and face-to-face administration methods. For the online component, questionnaires codes linked to the wenjuanxing platform were systematically distributed through officially verified WeChat groups managed by neighborhood committees, leveraging existing community networks to facilitate participation among older adults with technological proficiency. Simultaneously, trained researchers conducted home visits using mobile phones or tablet devices equipped with questionnaires code scanning functionality to perform face-to-face data collection. This modality specifically targeted older adults without personal smartphones or those with limited digital literacy, ensuring their inclusion through assisted participation.

#### 2.2.3 Statistical analysis

Data analysis was performed using IBM SPSS Statistics 24 (IBM, Inc., New York, USA) and R version 4.4.0 (2024-04-24) along with Z-stats 1.0. Shapiro–Wilk normality testing was performed on continuous variables (α = 0.05). Normally distributed data were summarized as mean ± standard deviation (SD), while non-normal data were reported as median with interquartile range (IQR). Categorical variables were described using frequencies and proportions (%). Rank-sum tests were employed for group comparisons of ordinal variables. Variables were initially screened using univariate binary logistic regression. Significant predictors were entered into a multivariable binary logistic regression model via forward stepwise selection (entry criterion: *p* < 0.05, likelihood ratio test). Model fit was assessed using the Hosmer-Lemeshow test (*p* > 0.05). Results presented both unadjusted odds ratios (OR) from univariate analysis and adjusted ORs from the multivariable model.

## 3 Results

### 3.1 The characteristics of participants

A total of 3,577 questionnaires were returned for this survey. After excluding 92 invalid responses due to incomplete entries or irregular response patterns, 3,485 valid responses were retained, yielding a response rate of 97.43%. Of these, 1,202 (34.49%) were completed by older adults (aged ≥60 years), which exceeds the predetermined threshold of 25.28% for subgroup analysis.

The 1,202 valid questionnaires from older adults had an average age of 65.5 years (SD 5.5 years), and 60.48% of them were aged 60–65 years. Of these, 661 (54.99%) were male and 541 (45.01%) were female. Of these respondents, 755 (62.81%) were urban residents and 447 (37.19%) were rural residents. Most of the respondents were married (81.28%), had a junior high school education or less (74.21%), and were farmers (56.49%). Most households had three to five members (62.56%), and most lived with their children (52.91%). Most had a monthly income of less than 3,000 RMB (70.97%). The details are shown in Table 1.

**Table 1:** Characteristics and comparison of DHL of the older adults.

Variables	Total (*n* = 1,202)	Qualified (*n* = 366)	Un-qualified (*n* = 836)	Statistic	*p*
Age, *n* (%)				Z = -5.47	**<0.001**
60 ~ 65	727 (60.48)	260 (35.76)	467 (64.24)		
66 ~ 70	277 (23.04)	80 (28.88)	197 (71.12)		
71 ~ 75	128 (10.65)	15 (11.72)	113 (88.28)		
76 ~ 80	49 (4.08)	8 (16.33)	41 (83.67)		
81and above	21 (1.75)	3 (14.29)	18 (85.71)		
Gender, *n* (%)				χ^2^ = 5.57	**0.018**
Male	661 (54.99)	220 (33.28)	441 (66.72)		
Female	541 (45.01)	146 (26.99)	395 (73.01)		
Location, *n* (%)				χ^2^ = 29.82	**<0.001**
Urban	755 (62.81)	272 (36.03)	483 (63.97)		
Rural	447 (37.19)	94 (21.03)	353 (78.97)		
Marital status, *n* (%)				χ^2^ = 11.36	**0.010**
Unmarried	9 (0.75)	2 (22.22)	7 (77.78)		
Married	977 (81.28)	310 (31.73)	667 (68.27)		
Divorced	56 (4.66)	22 (39.29)	34 (60.71)		
Widowed	160 (13.31)	32 (20.00)	128 (80.00)		
Ethnic group, *n* (%)				-^*^	0.304
Han nationality	1,201 (99.92)	365 (30.39)	836 (69.61)		
Ethnic minorities	1 (0.08)	1 (100.00)	0 (0.00)		
Education, *n* (%)				Z = -4.28	**<0.001**
Middle school and below	892 (74.21)	242 (27.13)	650 (72.87)		
High school or technical secondary school	257 (21.38)	101 (39.30)	156 (60.70)		
Associate degree	39 (3.24)	17 (43.59)	22 (56.41)		
Bachelor’s degree	12 (1.00)	6 (50.00)	6 (50.00)		
Master’s degree and above	2 (0.17)	0 (0.00)	2 (100.00)		
Occupation, *n* (%)				χ^2^ = 15.11	**<0.001**
Non-Farmer/worker	523 (43.51)	190 (36.33)	333 (63.67)		
Farmer/worker	679 (56.49)	176 (25.92)	503 (74.08)		
Family numbers, *n* (%)				χ^2^ = 3.05	0.218
< 3	278 (23.13)	84 (30.22)	194 (69.78)		
3 ~ 5	752 (62.56)	239 (31.78)	513 (68.22)		
> 5	172 (14.31)	43 (25.00)	129 (75.00)		
Domicile, *n* (%)				-	0.280
With relatives	636 (52.91)	202 (31.76)	434 (68.24)		
With spouse	463 (38.52)	129 (27.86)	334 (72.14)		
Alone	96 (7.99)	34 (35.42)	62 (64.58)		
Other	7 (0.58)	1 (14.29)	6 (85.71)		
Children’s concern about their health, *n* (%)				χ^2^ = 28.39	**<0.001**
Very concerned	687 (57.15)	250 (36.39)	437 (63.61)		
Somewhat concerned	337 (28.04)	82 (24.33)	255 (75.67)		
Neutral	147 (12.23)	29 (19.73)	118 (80.27)		
Not very concerned	18 (1.50)	3 (16.67)	15 (83.33)		
Not concerned at all	13 (1.08)	2 (15.38)	11 (84.62)		
Per capita monthly household income, *n* (%)				χ^2^ = 2.68	0.613
<500	56 (4.66)	20 (35.71)	36 (64.29)		
500 ~ 1,000	294 (24.46)	80 (27.21)	214 (72.79)		
1,001 ~ 3,000	503 (41.85)	154 (30.62)	349 (69.38)		
3,001 ~ 5,000	233 (19.38)	74 (31.76)	159 (68.24)		
v>5,000	116 (9.65)	38 (32.76)	78 (67.24)		
Medical insurance, *n* (%)				-	**<0.001**
UEBMI	396 (32.95)	151 (38.13)	245 (61.87)		
URRBMI	804 (66.89)	214 (26.62)	590 (73.38)		
Uninsured	2 (0.17)	1 (50.00)	1 (50.00)		
Monthly medical expenditures, *n* (%)				χ^2^ = 12.51	**0.006**
< 100	217 (18.05)	82 (37.79)	135 (62.21)		
100 ~ 500	669 (55.66)	178 (26.61)	491 (73.39)		
501 ~ 1,000	248 (20.63)	80 (32.26)	168 (7.74)		
> 1,000	68 (5.66)	26 (38.24)	42 (61.76)		
Healthcare Payment Methods, *n* (%)				-	**0.028**
URRBMI	773 (64.31)	230 (29.75)	543 (70.25)		
UEBMI	317 (26.37)	112 (35.33)	205 (64.67)		
Free medical treatment	3 (0.25)	1 (33.33)	2 (66.67)		
Commercial health Insurance	2 (0.17)	1 (50.00)	1 (50.00)		
Out-of-Pocket Payment	107 (8.90)	22 (20.56)	85 (79.44)		
Smoking, *n* (%)				χ^2^ = 1.02	0.313
Yes	402 (33.44)	130 (32.34)	272 (67.66)		
No	800 (66.56)	236 (29.50)	564 (70.50)		
Drinking, *n* (%)				χ^2^ = 1.60	0.206
Yes	399 (33.19)	131 (32.83)	268 (67.17)		
No	803 (66.81)	235 (29.27)	568 (70.73)		
Chronic disease, *n* (%)				χ^2^ = 18.27	**<0.001**
No	568 (47.25)	207 (36.44)	361 (63.56)		
Yes	634 (52.75)	159 (25.08)	475 (74.92)		
Health status, *n* (%)				Z = -8.43	**<0.001**
Very good	281 (23.38)	145 (51.60)	136 (48.40)		
Good	417 (34.69)	116 (27.82)	301 (72.18)		
Fair	401 (33.36)	89 (22.19)	312 (77.81)		
Poor	95 (7.90)	16 (16.84)	79 (83.16)		
Very poor	8 (0.67)	0 (0.00)	8 (100.00)		
Own digital device, *n* (%)				χ^2^ = 33.12	**<0.001**
Yes	984 (81.86)	335 (34.04)	649 (65.96)		
No	218 (18.14)	31 (14.22)	187 (85.78)		
Interest to internet health information, *n* (%)				χ^2^ = 169.66	**<0.001**
Yes	585 (48.67)	282 (48.21)	303 (51.79)		
No	617 (51.33)	84 (13.61)	533 (86.39)		
View online push notifications, *n* (%)				χ^2^ = 156.87	**<0.001**
Yes	598 (49.75)	282 (47.16)	316 (52.84)		
No	604 (50.25)	84 (13.91)	520 (86.09)		
Search for health information, *n* (%)				χ^2^ = 230.92	**<0.001**
Yes	449 (37.35)	254 (56.57)	195 (43.43)		
No	753 (62.65)	112 (14.87)	641 (85.13)		
Comment on or share health information, *n* (%)				Z = -15.81	**<0.001**
Frequently	183 (15.22)	133 (72.68)	50 (27.32)		
Sometimes	287 (23.88)	124 (43.21)	163 (56.79)		
Rarely	732 (60.90)	109 (14.89)	623 (85.11)		
Attitudes toward online health information, *n* (%)				Z = -2.46	**0.014**
Disbelieve	276 (22.96)	53 (19.20)	223 (S80.80)		
Somewhat believe	448 (37.27)	178 (39.73)	270 (60.27)		
Uncertain	372 (30.95)	77 (20.70)	295 (79.30)		
Believe	78 (6.49)	44 (56.41)	34 (43.59)		
Strongly believe	28 (2.33)	14 (50.00)	14 (50.00)		
Bookmark Function, *n* (%)				χ^2^ = 249.79	**<0.001**
Correct	304 (25.29)	175 (57.57)	129 (42.43)		
Incorrect	160 (13.31)	89 (55.62)	71 (44.38)		
Do not know	738 (61.40)	102 (13.82)	636 (86.18)		
Health Website Overview, *n* (%)				χ^2^ = 238.97	**<0.001**
Correct	215 (17.89)	121 (56.28)	94 (43.72)		
Incorrect	298 (24.79)	157 (52.68)	141 (47.32)		
Do not know	689 (57.32)	88 (12.77)	601 (87.23)		
Sources of health information, *n* (%)				χ^2^ = 143.21	**<0.001**
Social/Interpersonal	381 (31.70)	156 (40.94)	225 (59.06)		
Television	272 (22.63)	96 (35.29)	176 (64.71)		
Radio	37 (3.08)	15 (40.54)	22 (59.46)		
Web browser	124 (10.32)	66 (53.23)	58 (46.77)		
Do not know	388 (32.28)	33 (8.51)	355 (91.49)		
Distance to medical institutions, *n* (%)				Z = -491	**<0.001**
Within 1 km	459 (38.19)	181 (39.43)	278 (60.57)		
1 ~ 2 km	431 (35.86)	110 (25.52)	321 (74.48)		
2 ~ 3 km	210 (17.47)	50 (23.81)	160 (76.19)		
Over 3 km	102 (8.49)	25 (24.51)	77 (75.49)		
Willingness to use telemedicine services, *n* (%)				χ^2^ = 242.24	**<0.001**
Yes	372 (30.95)	228 (61.29)	144 (38.71)		
No	142 (11.81)	21 (14.79)	121 (85.21)		
Do not know	688 (57.24)	117 (17.01)	571 (82.99)		
Digital health literacy training, *n* (%)				χ^2^ = 156.91	**<0.001**
Never attended	438 (36.44)	43 (9.82)	395 (90.18)		
Family guidance	583 (48.50)	259 (44.43)	324 (55.57)		
Community training	91 (7.57)	21 (23.08)	70 (76.92)		
Senior university	90 (7.49)	43 (47.78)	47 (52.22)		
Training needs, *n* (%)				χ^2^ = 147.84	**<0.001**
Need	699 (58.15)	308 (44.06)	391 (55.94)		
No need	195 (16.22)	16 (8.21)	179 (91.79)		
Neutral	308 (25.62)	42 (13.64)	266 (86.36)		

^*^Fisher exact text; −, Fisher–Freeman–Halton test; χ^2^, Chi-square test; Z, Mann–Whitney U test; SD, standard deviation; UEBMI, Urban Employee Basic Medical Insurance; URRBMI, Urban and Rural Resident Basic Medical Insurance. Bold values indicate statistical significance (*p* < 0.05).

### 3.2 eHEALS score

The mean total eHEALS score was 22.30 ± 10.62 (mean ± standard deviation [SD]), with an average score of 2.79 (SD = 1.33). Only 30.45% (*n* = 366) of participants met the qualification threshold (total score ≥ 32). The average scores for the three dimensions of application, evaluation, and decision-making abilities regarding online health information and services were 2.78 ± 1.34, 2.80 ± 1.34, and 2.78 ± 1.35, respectively. Item 7 scored the highest (2.81 ± 1.35), and Item 4 scored the lowest (2.77 ± 1.37). The details are shown in Table 2. The Cronbach’s α coefficients for all dimensions were well above 0.90, indicating excellent reliability.

**Table 2:** Scores of eHEALS of the older adults.

Variables	Total (*n* = 1,202)	Qualified (*n* = 366)	Unqualified (*n* = 836)	Statistic (*t*-test)	*p*
Dimension1, Mean ± SD	11.13 ± 5.40	17.48 ± 1.87	8.35 ± 3.87	55.09	**<0.001**
Item 1. I know what health resources are available on the Internet, Mean ± SD	2.81 ± 1.38	4.38 ± 0.49	2.12 ± 1.03	51.69	**<0.001**
Item 2 I know where to find helpful health resources on the Internet, Mean ± SD	2.78 ± 1.37	4.37 ± 0.48	2.08 ± 0.99	54.02	**<0.001**
Item 3. I know how to use the Internet to answer my health questions, Mean ± SD	2.77 ± 1.35	4.35 ± 0.48	2.08 ± 0.98	53.72	**<0.001**
Item 4. I know how to use the health information I find on the Internet to help me, Mean ± SD	2.77 ± 1.37	4.37 ± 0.49	2.07 ± 0.99	53.75	**<0.001**
Item 5. I can tell high-quality health resources from low-quality ones on the Internet, Mean ± SD	2.79 ± 1.35	4.33 ± 0.49	2.11 ± 1.01	50.92	**<0.001**
Dimension2, Mean ± SD	5.58 ± 2.67	8.68 ± 0.94	4.23 ± 1.96	53.04	**<0.001**
Item 6. I feel confident in using information from the Internet to make health decisions, Mean ± SD	2.79 ± 1.34	4.35 ± 0.48	2.11 ± 0.99	52.65	**<0.001**
Item 7. I have the skills I need to evaluate the health resources I find on the Internet, Mean ± SD	2.81 ± 1.35	4.36 ± 0.48	2.13 ± 1.00	51.97	**<0.001**
Dimension3, Mean ± SD	2.78 ± 1.35	4.34 ± 0.52	2.09 ± 0.98	52.01	**<0.001**
Item 8. I feel confident in using information from the Internet to make health decisions, Mean ± SD	2.78 ± 1.35	4.34 ± 0.51	2.09 ± 0.98	52.01	**<0.001**
**Average score, Mean ± SD**	**2.79 ± 1.33**	**4.36 ± 0.46**	**2.10 ± 0.94**	**55.68**	**<0.001**
**Total score, Mean ± SD**	**22.30 ± 10.62**	**34.86 ± 3.67**	**16.80 ± 7.56**	**55.68**	**<0.001**

Bold values indicate statistical significance (*p* < 0.05).

### 3.3 Group comparisons

Comparisons between the two groups were made in terms of age, gender, residency, marital status, education level, occupation, children’s concern for their health, type of medical insurance, medical expenditure, payment method of medical expenses, chronic disease status, health status, presence or absence of electronic equipment, interest in online health knowledge, online health information behaviors (viewing and promoting messages, active retrieval, comment or forwarding), and online health information. Statistically significant differences were found in attitudes, online knowledge (e.g., knowledge of the bookmark function and health website overview), access to health science, distance from medical institutions, willingness to use telemedicine services, training experience, and training needs (*p* < 0.05). The details are presented in Table 1.

### 3.4 Predictors of digital health literacy

Binary logistic regression was conducted to identify associations between several factors and DHL among the older (see Table 3). The results revealed that gender, place of residence, and medical monthly expenditure were associated with DHL. Men had greater DHL than women (odds ratio [OR], 0.71; 95% confidence interval [CI], 0.51–0.99; *p* < 0.05). Older individuals living in urban areas had greater DHL than those living in rural areas (OR: 0.63; 95% CI: 0.43–0.92; *p* < 0.05). Those with an average monthly medical expenditure of 100–500 yuan were associated with lower DHL compared to the reference group (OR: 0.63; 95% CI: 0.40–0.99; p < 0.05). Although educational attainment showed a significant association with DHL in univariate analysis, this relationship was attenuated and lost statistical significance after adjustment for key sociodemographic and health-related variables, irrespective of whether education was classified into five categories (Table 3) or dichotomized as “middle school or below” versus “high school or above.”

**Table 3:** Analysis of Influencing Factors on DHL among the older.

Variables	Univariate binary logistic regression analysis					Multivariate binary logistic regression analysis				
	β	S.E	Z	OR (95%CI)	*p*	β	S.E	Z	OR (95%CI)	*p*
Age										
60 ~ 65				1.000 (Reference)					1.000 (Reference)	
66 ~ 70	−0.32	0.15	−2.06	0.73 (0.54 ~ 0.99)	**0.040**	0.17	0.21	0.80	1.18 (0.79 ~ 1.78)	0.421
71 ~ 75	−1.43	0.29	−5.02	0.24 (0.14 ~ 0.42)	**<0.001**	−0.42	0.36	−1.18	0.66 (0.32 ~ 1.32)	0.239
76 ~ 80	−1.05	0.39	−2.66	0.35 (0.16 ~ 0.76)	**0.008**	−0.17	0.54	−0.31	0.84 (0.29 ~ 2.45)	0.753
81 and above	−1.21	0.63	−1.92	0.30 (0.09 ~ 1.03)	0.055	−0.70	0.84	−0.84	0.50 (0.10 ~ 2.55)	0.401
Gender										
Male				1.000 (Reference)					1.000 (Reference)	
Female	−0.30	0.13	−2.36	0.74 (0.58 ~ 0.95)	**0.018**	−0.35	0.17	−2.05	0.71 (0.51 ~ 0.99)	**0.041**
Location										
Urban				1.000 (Reference)					1.000 (Reference)	
Rural	−0.75	0.14	−5.40	0.47 (0.36 ~ 0.62)	**<0.001**	−0.46	0.19	−2.41	0.63 (0.43 ~ 0.92)	**0.016**
Education										
Middle school and below				1.000 (Reference)					1.000 (Reference)	
High school or technical secondary school	0.55	0.15	3.73	1.74 (1.30 ~ 2.33)	**<0.001**	−0.13	0.21	−0.62	0.88 (0.58 ~ 1.32)	0.533
Associate degree	0.73	0.33	2.20	2.08 (1.08 ~ 3.98)	**0.028**	0.09	0.42	0.20	1.09 (0.47 ~ 2.50)	0.841
Bachelor’s degree	0.99	0.58	1.70	2.69 (0.86 ~ 8.41)	0.090	0.02	0.64	0.03	1.02 (0.29 ~ 3.55)	0.980
Master’s degree and above	−12.58	378.59	−0.03	0.00 (0.00 ~ Inf)	0.973	−14.85	761.92	−0.02	0.00 (0.00 ~ Inf)	0.984
Occupation										
Non-Farmer/worker				1.000 (Reference)					1.000 (Reference)	
Farmer/worker	−0.49	0.13	−3.87	0.61 (0.48 ~ 0.79)	**<0.001**	0.06	0.19	0.32	1.06 (0.73 ~ 1.55)	0.747
Medical insurance										
UEBMI				1.000 (Reference)					1.000 (Reference)	
URRBMI	−0.53	0.13	−4.06	0.59 (0.46 ~ 0.76)	**<0.001**	−0.35	0.19	−1.82	0.70 (0.48 ~ 1.03)	0.069
Uninsured	0.48	1.42	0.34	1.62 (0.10 ~ 26.13)	0.733	1.26	1.91	0.66	3.52 (0.08 ~ 149.21)	0.510
Children’s concern about their health										
Very concerned				1.000 (Reference)					1.000 (Reference)	
Somewhat concerned	−0.58	0.15	−3.85	0.56 (0.42 ~ 0.75)	**<0.001**	−0.25	0.20	−1.23	0.78 (0.52 ~ 1.16)	0.217
Neutral	−0.84	0.22	−3.81	0.43 (0.28 ~ 0.66)	**<0.001**	−0.34	0.30	−1.14	0.71 (0.39 ~ 1.28)	0.255
Not very concerned	−1.05	0.64	−1.65	0.35 (0.10 ~ 1.22)	0.099	−0.33	0.93	−0.35	0.72 (0.12 ~ 4.47)	0.725
Not concerned at all	−1.15	0.77	−1.48	0.32 (0.07 ~ 1.45)	0.138	0.27	1.03	0.26	1.31 (0.17 ~ 9.76)	0.795
Monthly medical expenditures										
< 100				1.000 (Reference)					1.000 (Reference)	
100 ~ 500	−0.52	0.17	−3.13	0.60 (0.43 ~ 0.82)	**0.002**	−0.47	0.23	−2.02	0.63 (0.40 ~ 0.99)	**0.044**
501 ~ 1,000	−0.24	0.20	−1.25	0.78 (0.53 ~ 1.15)	0.212	−0.15	0.28	−0.54	0.86 (0.50 ~ 1.48)	0.589
> 1,000	0.02	0.29	0.07	1.02 (0.58 ~ 1.79)	0.947	0.34	0.43	0.79	1.41 (0.60 ~ 3.28)	0.427
Chronic disease										
No				1.000 (Reference)					1.000 (Reference)	
Yes	−0.54	0.13	−4.26	0.58 (0.46 ~ 0.75)	**<0.001**	−0.15	0.18	−0.82	0.86 (0.61 ~ 1.23)	0.410
Health status										
Very good				1.000 (Reference)					1.000 (Reference)	
Good	−1.02	0.16	−6.29	0.36 (0.26 ~ 0.50)	**<0.001**	−0.34	0.22	−1.50	0.71 (0.46 ~ 1.11)	0.135
Fair	−1.32	0.17	−7.78	0.27 (0.19 ~ 0.37)	**<0.001**	−0.31	0.25	−1.24	0.73 (0.45 ~ 1.19)	0.213
Poor	−1.66	0.30	−5.55	0.19 (0.11 ~ 0.34)	**<0.001**	−0.43	0.42	−1.03	0.65 (0.29 ~ 1.47)	0.301
Very poor	−14.63	312.10	−0.05	0.00 (0.00 ~ Inf)	0.963	−14.32	445.55	−0.03	0.00 (0.00 ~ Inf)	0.974
Own digital device										
Yes				1.000 (Reference)					1.000 (Reference)	
No	−1.14	0.21	−5.53	0.32 (0.21 ~ 0.48)	**<0.001**	−0.32	0.30	−1.06	0.73 (0.41 ~ 1.31)	0.289
Interest to internet health information										
Yes				1.000 (Reference)					1.000 (Reference)	
No	−1.78	0.14	−12.36	0.17 (0.13 ~ 0.22)	**<0.001**	−0.24	0.25	−0.93	0.79 (0.48 ~ 1.30)	0.350
View online push notifications										
Yes				1.000 (Reference)					1.000 (Reference)	
No	−1.71	0.14	−11.93	0.18 (0.14 ~ 0.24)	**<0.001**	0.21	0.27	0.76	1.23 (0.72 ~ 2.11)	0.445
Search for health information										
Yes				1.000 (Reference)					1.000 (Reference)	
No	−2.01	0.14	−14.37	0.13 (0.10 ~ 0.18)	**<0.001**	−0.39	0.25	−1.57	0.68 (0.42 ~ 1.10)	0.116
Comment on or share health information										
Frequently				1.000 (Reference)					1.000 (Reference)	
Sometimes	−1.25	0.20	−6.13	0.29 (0.19 ~ 0.43)	**<0.001**	−0.52	0.25	−2.06	0.60 (0.36 ~ 0.98)	**0.040**
Rarely	−2.72	0.20	−13.91	0.07 (0.04 ~ 0.10)	**<0.001**	−0.71	0.28	−2.54	0.49 (0.28 ~ 0.85)	**0.011**
Attitudes toward online health information										
Disbelieve				1.000 (Reference)					1.000 (Reference)	
Somewhat believe	1.02	0.18	5.64	2.77 (1.95 ~ 3.95)	**<0.001**	0.43	0.26	1.67	1.53 (0.93 ~ 2.52)	0.095
Uncertain	0.09	0.20	0.47	1.10 (0.74 ~ 1.62)	0.638	0.09	0.27	0.33	1.09 (0.65 ~ 1.84)	0.742
Believe	1.69	0.27	6.17	5.45 (3.18 ~ 9.33)	**<0.001**	0.86	0.37	2.35	2.37 (1.15 ~ 4.86)	**0.019**
Strongly believe	1.44	0.41	3.52	4.21 (1.89 ~ 9.36)	**<0.001**	1.92	0.57	3.37	6.80 (2.23 ~ 20.77)	**<0.001**
Bookmark Function										
Correct				1.000 (Reference)					1.000 (Reference)	
Incorrect	−0.08	0.20	−0.40	0.92 (0.63 ~ 1.36)	0.688	−0.14	0.25	−0.57	0.87 (0.53 ~ 1.42)	0.570
Do not know	−2.14	0.16	−13.55	0.12 (0.09 ~ 0.16)	**<0.001**	−0.59	0.24	−2.47	0.56 (0.35 ~ 0.89)	**0.013**
Health Website Overview										
Correct				1.000 (Reference)					1.000 (Reference)	
Incorrect	−0.15	0.18	−0.81	0.87 (0.61 ~ 1.23)	0.420	0.03	0.23	0.13	1.03 (0.66 ~ 1.61)	0.893
Do not know	−2.17	0.18	−12.16	0.11 (0.08 ~ 0.16)	**<0.001**	−0.37	0.26	−1.42	0.69 (0.41 ~ 1.15)	0.155
Sources of health information										
Social/Interpersonal				1.000 (Reference)					1.000 (Reference)	
Television	−0.24	0.16	−1.46	0.79 (0.57 ~ 1.09)	0.144	−0.36	0.22	−1.64	0.70 (0.46 ~ 1.07)	0.101
Radio	−0.02	0.35	−0.05	0.98 (0.49 ~ 1.96)	0.962	−0.55	0.46	−1.21	0.58 (0.24 ~ 1.41)	0.228
Web browser	0.50	0.21	2.38	1.64 (1.09 ~ 2.47)	**0.017**	0.01	0.27	0.03	1.01 (0.59 ~ 1.72)	0.977
Do not know	−2.01	0.21	−9.58	0.13 (0.09 ~ 0.20)	**<0.001**	−0.46	0.29	−1.59	0.63 (0.36 ~ 1.11)	0.113
Distance to medical institutions										
Within 1 km				1.000 (Reference)					1.000 (Reference)	
1 ~ 2 km	−0.64	0.15	−4.39	0.53 (0.40 ~ 0.70)	**<0.001**	−0.33	0.19	−1.69	0.72 (0.49 ~ 1.05)	0.090
2 ~ 3 km	−0.73	0.19	−3.90	0.48 (0.33 ~ 0.69)	**<0.001**	−0.08	0.25	−0.34	0.92 (0.56 ~ 1.50)	0.735
Over 3 km	−0.70	0.25	−2.79	0.50 (0.31 ~ 0.81)	**0.005**	0.23	0.35	0.65	1.25 (0.63 ~ 2.49)	0.517
Willingness to use telemedicine services										
Yes				1.000 (Reference)					1.000 (Reference)	
No	−2.21	0.26	−8.53	0.11 (0.07 ~ 0.18)	**<0.001**	−1.06	0.32	−3.27	0.35 (0.18 ~ 0.66)	**0.001**
Do not know	−2.04	0.15	−13.90	0.13 (0.10 ~ 0.17)	**<0.001**	−0.64	0.20	−3.17	0.53 (0.35 ~ 0.78)	**0.002**
Digital health literacy training										
Never attended				1.000 (Reference)					1.000 (Reference)	
Family guidance	1.99	0.18	11.02	7.34 (5.15 ~ 10.47)	**<0.001**	0.88	0.24	3.66	2.41 (1.50 ~ 3.85)	**<0.001**
Community training	1.01	0.30	3.42	2.76 (1.54 ~ 4.92)	**<0.001**	0.80	0.35	2.26	2.23 (1.11 ~ 4.46)	**0.024**
Senior university	2.13	0.27	8.03	8.40 (5.00 ~ 14.13)	**<0.001**	1.30	0.35	3.72	3.67 (1.85 ~ 7.28)	**<0.001**
Training needs										
Need				1.000 (Reference)					1.000 (Reference)	
No need	−2.18	0.27	−8.01	0.11 (0.07 ~ 0.19)	**<0.001**	−0.54	0.35	−1.56	0.58 (0.29 ~ 1.15)	0.120
Neutral	−1.61	0.18	−8.80	0.20 (0.14 ~ 0.29)	**<0.001**	−0.56	0.23	−2.44	0.57 (0.37 ~ 0.90)	**0.015**

S.E, Standard Error; OR, Odds Ratio; CI, Confidence Interval; UEBMI, Urban Employee Basic Medical Insurance; URRBMI, Urban and Rural Resident Basic Medical Insurance. Bold values indicate statistical significance (*p* < 0.05).

Additionally, the frequency of network interaction, basic internet knowledge, attitudes toward internet health information, and willingness to use telemedicine services were statistically correlated with the DHL of older individuals. Older individuals who frequently comment or share health information online were more likely to have good DHL than those who sometimes (OR 0.60, 95%CI 0.36–0.98; *p* < 0.05) or rarely (OR 0.49, 95% CI 0.28–0.85; p < 0.05) interact online with others. A positive attitude toward internet health information was associated with DHL. Participants who believed in internet health information were 2.37 times more likely to have qualified DHL than those who did not believe in it (OR 2.37, 95% CI 1.15–4.86; *p* < 0.05). Older individuals who knew how to use the “Bookmark” function were more likely to have good DHL than those who did not (OR 0.56, 95% CI 0.35–0.89; *p* < 0.05). Additionally, older adults who were willing to use telemedicine services were more likely to have higher DHL than those who were unwilling (OR: 0.35; 95% CI: 0.18–0.66; *p* < 0.05) or indifferent (OR: 0.53; 95% CI: 0.35–0.78; p < 0.05).

Finally, training needs and training experiences were significantly associated with DHL among the older. Those with digital health-related training needs had greater DHL than those who were indifferent (OR 0.57, 95% CI 0.37–0.90; *p* < 0.05). Those who experienced digital health-related training were 2.23 to 3.67 times more likely to have qualified DHL than those who did not receive training from family members (OR 2.41, 95% CI 1.50–3.85; *p* < 0.05), community programs (OR 2.23, 95% CI 1.11–4.46; *p* < 0.01), or senior colleges (OR 3.67, 95% CI 1.85–7.28; *p* < 0.01), after adjusting for sociodemographic and other covariates.

## 4 Discussion

### 4.1 Principal findings

This study aimed to investigate the prevalence of DHL and associated factors among older individuals in Sichuan Province, China. Our results revealed that the total DHL score among the older adults was 22.30 (Table 2), which was much lower than the passing level (≥ 32), suggesting that more attention should be paid to the DHL of older adults. The results were similar to the results of a recent systematic review and meta-analysis with the average score of 21.45 (95% CI: 19.81–23.08) (38). However, older adults have significantly lower DHL than other groups, such as students (40–42) and non-older individuals (43). There are also differences in DHL levels among older adults of different nationalities, regions, and characteristics. In a cross-sectional survey of 2,144 older adults aged in China, the rate of adequate eHealth literacy was 11.9% (24), and the level of DHL in the older population was 2.16 (average score) in Jinan, China (44), which may be related to the older average age of the older individuals in this study and the high prevalence of mild cognitive impairment (16%, higher than the national average of 14%). A cross-sectional survey of two urban cities in South Korea (29) revealed that 22.3% of the participants had high eHealth literacy skills. Owing to the eligibility criteria, the participants were older adults aged 65 years with a mean age of 76.8 years. Participants aged 80 years and older accounted for 30% of the total sample. However, in our study, the average age was 65.5 years with an SD of 5.5 years. The most recent meta-analysis revealed that age is a key factor in higher eHealth literacy (β = −0.042, 95% CI -0.071 to −0.020) (45). While our study did not find a significant association between age and DHL by the result of multivariate binary logistic regression analysis. This discrepancy may be attributed to the underrepresentation of the oldest-old population in our sample, as participants aged 81 years and older accounted for only 1.75% (21/1202) of total sample (Table 1). The limited representation of this age group, who typically experience the steepest decline in digital literacy, may have reduced our ability to detect a significant age effect.

In this study, the average scores for the three dimensions (application ability, judgment ability, and decision-making ability) were all lower than three points, suggesting systemic weaknesses in health information retrieval, credibility identification, and health decision-making among the older. Item 4 (*I know how to use the health information I find online to help me*) had the lowest score, reflecting significant challenges faced by the older in the information screening and integration process. The lower DHL in our study may be related to the fact that the majority (74.21%) of the sample had an education level of junior high school or below. In previous studies (25, 39, 46–48), educational levels associated with DHL, while this association was attenuated and no longer statistically significant after adjustment for other sociodemographic and health-related factors. This suggests that the influence of education observed in univariate analysis may be largely mediated through its strong correlation with socioeconomic status and overall health. In this study, the effect of educational attainment is likely intertwined with generational experiences and broader life-course advantages, which are captured by other variables in the model. This finding may highlight the complex, indirect pathway through which education may influence DHL in older adults. Additionally, the difference in eligibility rates between urban (62.81%) and rural (37.19%) areas suggests that geographical distribution, infrastructure coverage, and access to educational resources may be key constraints (9).

### 4.2 The digital health literacy of older people is affected by several factors

DHL is contingent upon a multitude of factors, including sociodemographic, economic, and cultural elements (48, 49). As indicated by prior studies, older individuals, those with lower educational attainment, and infrequent internet users have been shown to have diminished DHL skills (50). In the present study, the DHL of older adults were found to be associated with their demographic characteristics, internet behaviors, personal attitudes toward education, and training experience. In terms of demographic characteristics, male and urban older people have been shown to have high levels of DHL (Table 3). This finding has been replicated in several articles. Males have been shown to have a positive correlation between high digital health literacy and internet use (17), In China, male older adults have been shown to have a higher level of education (51), which may affect their DHL level. Spanakis et al. reported that higher literacy levels were associated with self-reported internet knowledge that was considered to be “outstanding” or “good” (52). And product complexity and reliability, awareness of resources, lack of trust, and cost are common barriers to the use of digital health technologies for people from rural and regional areas (53). In the context of medical expenditures, a correlation has been observed between expenditures ranging from 100 to 500 yuan per month and diminished digital health literacy. Conversely, medical expenditures exceeding 500 yuan per month have been associated with a heightened propensity to seek online health information, driven by the prevailing notion that “long illness becomes good doctor.”

As indicated by the extant literature, a robust correlation exists between DHL and both the extent of one’s knowledge of the internet, as well as one’s internet usage behaviors (54, 55). Older individuals who possess fundamental internet proficiency and frequently contribute to or disseminate internet health information demonstrate elevated levels of DHL. This finding aligns with the observations reported by Zhao, who noted a positive correlation between the frequency of health-related internet usage and DHL among study participants (43). Furthermore, attitudes toward training and the extent of prior training experience have been identified as significant factors influencing the DHL levels of the older. In contrast, older individuals who exhibit a lack of necessity or ambivalence towards training demonstrate a reduced capacity in this regard. In comparison with those who have not undergone any form of training related to the internet, individuals who have participated in any type of training, such as guidance from family members, community training, or college training for the older, are associated with higher levels of DHL (Table 3). Liu et al. reported a positive correlation between the frequency of passive guidance received from family members and DHL in older adults (25). The propensity to utilize telemedicine services is also associated with DHL. However, cross-sectional studies are not equipped to confirm or invalidate a causal relationship, and it is challenging to ascertain whether the level of DHL affects the propensity to use the internet or whether the propensity to use the internet exerts an influence on the level of DHL.

### 4.3 Suggestions for improving the digital health literacy of older people

In light of the pivotal role of DHL in contemporary health management, the implementation of pragmatic and efficacious intervention strategies is imperative to augment DHL and the capacity to leverage information technology (56). However, the extant literature regarding DHL interventions remains limited, with the majority of research conducted in developed countries such as the USA (8, 57). A meta-analysis (8) including 7 studies with 710 older adults to assess the effectiveness of DHL interventions for older adults founded that considerable gains in knowledge (SMD 0.93, 95% CI 0.54–1.31; *p* < 0.001) and self-efficacy (SMD 0.96, 95% CI 0.16–1.77; *p* = 0.02) were observed, and concluded that DHL interventions have positive effects on the health status and health management of older adults. Intervention strategies encompass collaborative learning and customized interventions (58). Collaborative learning is defined as the construction of meaning through interactions with others (58, 59). Tailored interventions are usually used for specific individuals, such as older patients with hypertension or other health-related problems (60). The intervention’s contents encompass a wide range of subjects, including fundamental computer skills, prominent search engines, official patient portals, and peer support forums, among others (20). The intervention methods generally involve digital and onsite education or training, including online sessions, e-learning content, small class programs, and coaching involving smart devices and personal health records (57, 61).

It is important to note that DHL in the older is influenced by more than just their own digital competence. Social support and environmental circumstances also play a significant role. In the early 2017, a study revealed that socioeconomic status and community-level resources are associated with the digital divide of the older population in China (9). A study examined the effects of social support on video telehealth utilization among older Veterans and found that 32.2% of respondents noted that the absence of family or friends to assist with video visits hindered their use of video telehealth, that greater tangible social support was associated with 54.1% (95% CI: 10.1–116.2%) greater odds of having a video visit, and that assessing and addressing patients’ social and environmental circumstances may help optimize digital divide interventions (62). In patients with diabetes, family members may serve as facilitators, helping to bridge the so-called “digital divide” in health information technology. This can be achieved by assisting adults in accessing patient web portals or health information technology designed for diabetes management (63). Therefore, optimal strategies in the realm of digital health literacy must prioritize the delivery of experiences that are customized to individual needs, interactive, and action-oriented (64). These experiences should be further enriched by the consideration of socioenvironmental factors, such as family, and the integration of social support systems to facilitate lifelong eHealth learning for older adults (65). It is imperative to leverage the diverse roles of stakeholders, including families, communities, and governments, to ensure the effectiveness of these strategies.

Based on the findings of this study, the following targeted interventions are proposed to improve DHL among older adults. Firstly, regionally tailored programs will be established, providing fundamental operational training in rural areas while offering advanced digital health evaluation in urban settings. Additionally, gender-specific barriers will be addressed through women-focused digital clinics that incorporate peer learning groups. To enhance accessibility, multi-lingual visual guides and video tutorials will be distributed through primary healthcare institutions. Furthermore, family-digital-assistant programs will be developed to foster intergenerational support and build trust in digital health technologies. To address economic barriers, subsidized internet plans and smart devices will be provided through collaborations with telecommunications companies. These interventions will be implemented through collaborative efforts between healthcare institutions, community organizations, and technology providers.

### 4.4 Limitations

The present study is not without its limitations. Initially, the study concentrated on Pengzhou, located in Sichuan Province. However, it is important to note that the findings are not necessarily representative of the broader older population in China. The subsequent phase of this research will entail the execution of a nationwide multicenter survey, employing a substantial sample size. Secondly, the cross-sectional design of the study precludes the ability to draw causal conclusions. Unmeasured confounders, such as detailed cognitive assessments beyond mild impairment prevalence, may influence the results. Third, the survey methodology predominantly included older adults with pre-existing digital literacy skills, which may have introduced selection bias and potentially led to an overestimation of DHL levels in the broader older adult population. Furthermore, the collection of self-reported data is susceptible to recall bias, and while the urban–rural digital divide is acknowledged, it necessitates a more profound examination of its contextual underpinnings. The recommendation is for future multicentre longitudinal studies to address these gaps.

## 5 Conclusion

The study reveals that older adults in Sichuan reveals that their DHL is suboptimal. This phenomenon is influenced by a variety of factors, including demographics, internet behaviors, attitudes, and training. It is imperative to fortify educational and training programs, and multilevel interventions involving families, communities, and policymakers are indispensable to bridge the digital health gap.

## Data Availability

The raw data supporting the conclusions of this article will be made available by the authors, without undue reservation.
