# Supplementary material for: Digital health literacy among older adults in China: a cross-sectional study on prevalence and influencing factors
Source: Front Public Health. 2025 Sep 23;13:1661177. doi: 10.3389/fpubh.2025.1661177 (PMC12500426; doi:10.3389/fpubh.2025.1661177)
Supplement: Supplementary file 1 [file Data_Sheet_1.pdf]

Supplementary materials:

1. Questionnaire on Health Status and Digital Health Literacy Among Urban and Rural Residents (Chinese);
2. Supplementary Table 1. Sample Selection for Survey;
3. Supplementary Table 2. Inter-item Correlation Matrix

Questionnaire on Health Status and Digital Health Literacy Among Urban and Rural Residents

Dear Madam/Sir,

Hello! Thank you very much for taking the time to complete this questionnaire. We are a research team from West China Hospital of Sichuan University. The purpose of this survey is to understand the health status, disease burden, and level of eHealth literacy among urban and rural residents in this city.

We assure you that: The results of this survey will be used for research purposes only. All information you provide will be kept strictly confidential. Your participation will not have any negative impact on you.

Please answer the questions honestly and rest assured. Thank you for your participation!

Part 1: Basic Information

1. Your age: (   ) [Single choice] *

A. Under 18

B. 18–30

C. 31–45

D. 46–60

E. 60 and above

2. Your gender: (   ) [Single choice] *

A. Male

B. Female

3. Your place of residence: (   ) [Fill-in-the-blank] *

____

4. Your marital status: (   ) [Single choice] *

A. Unmarried

B. Married

C. Divorced

D. Widowed

5. Your ethnicity: (   ) [Single choice] *

A. Han Chinese

B. Ethnic minority

6. Your education level: (   ) [Single choice] *

A. Junior high school or below

B. High school / vocational school / technical secondary school

C. Associate degree

D. Bachelor’s degree

E. Master’s degree or above

7. Your occupation: (   ) [Single choice] *

A. Farmer / farm worker

B. Non-farmer / migrant worker

8. Your basic medical insurance type: (   ) [Single choice] *

A. Basic medical insurance for employees

B. Basic medical insurance for urban and rural residents

C. Not enrolled (please specify reason) _________________ *

9. Number of people in your household: (　　) [Single choice] *

A. Fewer than 3

B. 3–5

C. More than 5

10. Your household's monthly income per capita: (　　) [Single choice] *

A. Under ¥500

B. ¥500–1,000

C. ¥1,001–3,000

D. ¥3,001–5,000

E. Above ¥5,000

11. Your household's monthly medical expenses: (　　) [Single choice] *

A. Under ¥100

B. ¥100–500

C. ¥500–1,000

D. Above ¥1,000

12. Main payment method for your medical expenses: (　　) [Single choice] *

A. Basic medical insurance for urban and rural residents

B. Basic medical insurance for employees

C. Government-covered medical service

D. Commercial health insurance

E. Fully self-paid

13. Your smoking status: (　　) [Single choice] *

A. Smoker

B. Non-smoker

14. Your drinking status: (   ) [Single choice] *

A. Drinker

B. Non-drinker

15. Do you currently have any of the following chronic diseases? (   ) [Single choice] *

A. No chronic disease

B. Hypertension

C. Heart disease

D. Cerebrovascular disease (e.g., stroke, cerebral infarction, thrombosis)

E. Diabetes

F. Malignant tumor

G. Other _________________ * (If “Other,” please specify)

16. Over the past year, how would you rate your health status? (   ) [Single choice] *

A. Excellent

B. Good

C. Fair

D. Poor

E. Very poor

17. In the past year, what were the main diseases or reasons for you or your family members seeking medical care? (   ) [Multiple choice] *

A. Common cold

B. Health check-up

C. Hypertension, hyperlipidemia, hyperglycemia

D. Coronary heart disease, congenital heart disease, or other heart diseases

E. Bone and joint disease

F. Pneumoconiosis

G. Chronic bronchitis

H. Cerebrovascular disease

I. Rheumatoid arthritis

J. Acute or chronic gastritis

K. Various types of cancer

L. Other _________________ * (If "Other," please specify)

Part 2: eHealth Literacy Survey

Concept Explanation: eHealth literacy refers to an individual's ability to search, filter, understand, evaluate, and apply health information obtained from electronic resource platforms to solve health-related problems.

(I) Basic Situation

1. Do you own electronic devices such as a mobile phone or computer?
(   ) [Single choice] *

A. Yes

B. No

2. Are you interested in learning health-related knowledge or skills online?
(   ) [Single choice] *

A. Yes

B. No

3. Do you check health information pushed by online sources? (　　) [Single choice] *

A. Yes

B. No

4. Do you often actively search for disease-related information online? (　　) [Single choice] *

A. Yes

B. No

5. Which channels do you usually use to search for disease information online? (　　) [Multiple choice] *

A. Search engines (e.g., Baidu)

B. Social platforms (e.g., QQ, WeChat, Weibo, Douyin, Xiaohongshu)

C. Official websites of medical institutions or health departments

D. Other ________________ (If "Other," please specify)

6. Do you forward or share online health-related information? (　　) [Single choice] *

A. Often

B. Sometimes

C. Rarely

7. What is your attitude toward online health information? (　　) [Single choice] *

A. Disbelieve

B. Somewhat believe

C. Uncertain

D. Believe

E. Strongly believe

8. The function of the “Favorites” in a web browser is: (　　) [Single choice] *

A. To save certain web addresses for easy future access

B. To copy content from a webpage

C. To print content from a webpage

D. To hide content on a webpage

E. I don’t know

9. If you want to quickly access some health education information, the best way is: (　　) [Single choice] *

A. Social and interpersonal channels (e.g., asking friends)

B. Television

C. Radio

D. Searching via a web browser

E. I don’t know

10. If you want to learn about the introduction of a health website (e.g., Dingxiangyuan), which section should you check? (　　) [Single choice] *

A. About Us

B. Services

C. Friends Links

D. Contact Us

E. I don’t know

11. The distance from your home to the nearest community health service center / clinic is: (   ) [Single choice] *

A. Within 1 km

B. 1–2 km

C. 2–3 km

D. >3 km

12. Would you choose to use online consultation or other telemedicine services? (   ) [Single choice] *

A. Yes

B. No ________________ (If “No,” please specify the reason)

C. Not sure

13. eHealth Literacy Questionnaire (eHEALS) (Please select the option that matches your situation) [Matrix question] *

Item	Strongly agree	Agree	Unsure	Disagree	Strongly disagree
I know how to find helpful health resources on the internet	○	○	○	○	○
I know how to use the internet to answer my health questions	○	○	○	○	○

Item	Strongly agree	Agree	Unsure	Disagree	Strongly disagree
I know what health resources are available on the internet	○	○	○	○	○
I know where to find helpful health resources on the internet	○	○	○	○	○
I know how to use the health information I find on the internet to help myself	○	○	○	○	○
I have the skills to evaluate the quality of health resources on the internet	○	○	○	○	○
I can tell high-quality health resources from low-quality ones on the internet	○	○	○	○	○
I feel confident in using information from the Internet to make health decisions	○	○	○	○	○

Supplementary Table 1. Sample Selection for Survey

**Order**	**Region**	**Resident Population**	**Percentage (%)**	**Planned Sample Size**
1	Street A	231867	29.71%	865
2	Street B	105426	13.51%	394
3	Street C	108801	13.94%	406
4	Street D	55026	7.05%	205
5	Town A	58538	7.50%	219
6	Town B	49254	6.31%	184
7	Town C	34189	4.38%	128
8	Town D	16922	2.17%	63
9	Town E	26448	3.39%	99
10	Town F	8470	1.09%	32
11	Town G	23596	3.02%	88
12	Town F	27451	3.52%	102
13	Town H	34411	4.41%	128
Total		780399	100.00%	2913

Supplementary Table 2. Inter-item Correlation Matrix

	Dimension 1	Dimension 2	Dimension 3
Dimension 1	1.000	0.960	0.948
Dimension 2	0.960	1.000	0.977
Dimension 3	0.948	0.977	1.000
